# Molecular mechanism of protrusion formation during cell-to-cell spread of *Listeria*

**DOI:** 10.3389/fcimb.2014.00021

**Published:** 2014-02-21

**Authors:** Keith Ireton, Luciano A. Rigano, Lilia Polle, Wolf-Dieter Schubert

**Affiliations:** ^1^Department of Microbiology and Immunology, University of OtagoDunedin, New Zealand; ^2^Department of Biotechnology, University of the Western CapeBellville, Cape Town, South Africa; ^3^Department of Biochemistry, University of PretoriaPretoria, South Africa

**Keywords:** *Listeria monocytogenes*, cell-to-cell spread, protrusion, InlC, Tuba, SH3 domain, cortical tension, structural elucidation

## Abstract

The bacterial pathogen *Listeria monocytogenes* spreads within human tissues using a motility process dependent on the host actin cytoskeleton. Cell-to-cell spread involves the ability of motile bacteria to remodel the host plasma membrane into protrusions, which are internalized by neighboring cells. Recent results indicate that formation of *Listeria* protrusions in polarized human cells involves bacterial antagonism of a host signaling pathway comprised of the scaffolding protein Tuba and its effectors N-WASP and Cdc42. These three human proteins form a complex that generates tension at apical cell junctions. *Listeria* relieves this tension and facilitates protrusion formation by secreting a protein called InlC. InlC interacts with a Src Homology 3 (SH3) domain in Tuba, thereby displacing N-WASP from this domain. Interaction of InlC with Tuba is needed for efficient *Listeria* spread in cultured human cells and infected animals. Recent structural data has elucidated the mechanistic details of InlC/Tuba interaction, revealing that InlC and N-WASP compete for partly overlapping binding surfaces in the Tuba SH3 domain. InlC binds this domain with higher affinity than N-WASP, explaining how InlC is able to disrupt Tuba/N-WASP complexes.

## Introduction

**Figure 1:**
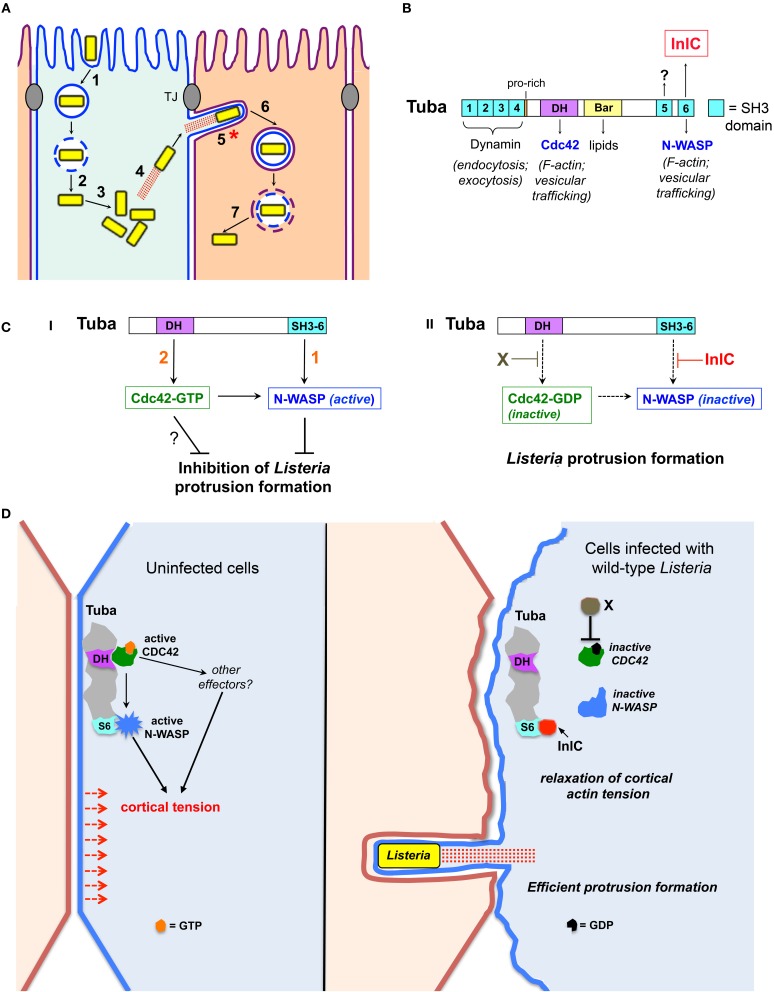
**Bacterial and host factors controlling protrusion formation in polarized epithelial cells. (A)** Steps in the intracellular life cycle of *Listeria*. (1) internalization of bacteria into host cells, (2) escape from phagosomes, (3) bacterial replication in the cytosol, (4) actin based motility, (5) formation of bacterial protrusions, (6) engulfment of protrusions, and (7) dissolution of the double membranous vacuole. The process of cell–cell spread comprises steps 4–7. This minireview focuses on molecular events controlling protrusion formation (indicated with an asterisk). “TJ” denotes tight junctions. **(B)** Domain structure of the human scaffolding protein Tuba. SH3 domains (light blue) are numbered 1–6. SH3 domains 1–4 bind the GTPase Dynamin, whereas SH3 domain 6 (SH3-6) interacts with N-WASP. SH3-6 is also targeted by the secreted listerial protein InlC. Ligands of SH3-5 are unknown. The Dbl Homology (DH) domain has guanine nucleotide exchange activity for the human GTPase Cdc42. The Bar domain presumably binds lipids of the plasma membrane inner leaflet. Physiological processes controlled by Tuba ligands are listed. **(C)** Relief of Tuba-mediated inhibition of *Listeria* spreading by InlC and other bacterial factors: (i) Without InlC or other bacterial proteins, listerial protrusion formation is restrained by [1] Tuba SH3-6 binding to N-WASP to recruit N-WASP to the plasma membrane and/or activate N-WASP, and [2] Tuba-mediated stimulation of Cdc42 to impair protrusions by activating N-WASP and/or other effectors (?). (ii). InlC relieves Tuba-mediated inhibition of bacterial spread by blocking N-WASP binding to Tuba SH3-6. In addition, an unknown bacterial factor “X” promotes protrusion formation by reducing Cdc42 activity. **(D)** Model of tight junction perturbation by InlC. In uninfected polarized epithelial cells or cells infected with Δ*inlC* bacteria (left panel), host Tuba, N-WASP and Cdc42 together promote cortical tension presumably via Tuba-mediated activation of Cdc42 and of N-WASP as well as Cdc42-GTP activation of N-WASP and possibly of additional effectors. In cells infected with wild-type *Listeria* (right panel), cortical tension is diminished by InlC displacing N-WASP from Tuba SH3-6 and by Cdc42 inhibition via an unidentified bacterial factor “X.” Reduced tension allows efficient protrusion formation by *Listeria*.

Several bacteria, including the enteric pathogens *Listeria monocytogenes* and *Shigella flexneri* and select species of the arthopod-borne genus *Rickettsia*, use an actin-based motility process to actively spread within human tissues (Gouin et al., 2005; Haglund and Welch, 2011; Ireton, 2013). After internalization into human cells, bacteria induce the lysis of host-derived membrane vacuoles, and subsequently replicate in the cytoplasm (Figure 1A; steps 1–3). Cytosolic bacteria stimulate the assembly of actin filaments, resulting in intracellular motility (step 4). *Listeria* and *Shigella* have mechanisms to evade autophagy- a host degradative pathway that would other otherwise kill cytoplasmic microbes (Cemma and Brumell, 2012; Mostowy and Cossart, 2012). Bacteria propelled by actin-based motility ultimately contact and remodel the host plasma membrane into thin membrane projections termed “protrusions” (Figure 1A, step 5). These protrusions are internalized by neighboring cells, resulting in microbes in double-membranous vacuoles (step 6). Finally, the vacuoles are destroyed by bacterial factors, liberating bacteria into the cytosol of the newly infected cell.

Much progress has been made in understanding the mechanisms of F-actin assembly by *Listeria*, *Shigella*, and *Rickettsia* (Gouin et al., 2005; Haglund and Welch, 2011; Ireton, 2013). In brief, the *Listeria* surface protein ActA and *Shigella* protein IcsA each activate a mammalian actin polymerization machinery called the Arp2/3 complex. ActA is a direct activator (“nucleation promoting factor”) of Arp2/3, whereas IcsA stimulates Arp2/3 through recruitment of a human nucleation promoting factor known as N-WASP. Unlike ActA or IcsA, the *Rickettsia* surface protein Sca2 directly promotes assembly of actin filaments independently of Arp2/3 or other host factors. Sca2 may act as a functional mimic of a class of eukaryotic actin polymerization proteins called formins. Compared to the fairly detailed understanding of F-actin assembly by *Listeria*, *Shigella*, and *Rickettsia*, much less is know about how these pathogens produce protrusions that mediate cell-to-cell spread. This mini-review focuses on recent studies with *Listeria* that have shed light on the molecular mechanism of protrusion formation.

## *Listeria* protrusions are controlled by the bacterial protein InlC and its host ligand Tuba

Recent work led to the identification of microbial and human proteins that regulate protrusion formation by *Listeria* (Rajabian et al., 2009). A bacterial mutant deleted for the gene *inlC* (Δ*inlC*) was found to be partly defective in in cell-to-cell spread in the polarized human cell line Caco-2 BBE1. Confocal microscopy analysis indicated that the Δ*inlC* mutant is unaffected in phagosomal escape or in the ability to produce F-actin tails. Instead the mutant is compromised in the formation of protrusions, producing these structures at only about 50% the efficiency of the wild-type bacterial strain.

InlC is a member of the internalin family of *Listeria* proteins (Engelbrecht et al., 1996). These proteins share a common architecture including an amino terminal cap domain, a leucine-rich-repeat (LRR) domain that often mediates interaction with host proteins and a immunoglobulin-like interrepeat domain (Schubert et al., 2001; Bierne et al., 2007). Interestingly, although most internalin proteins are anchored to the *Listeria* cell surface, InlC is entirely secreted (Engelbrecht et al., 1996). Importantly, the expression of *inlC* is greatly increased upon internalization of bacteria into human cells due to the action of the bacterial transcription factor PrfA (Engelbrecht et al., 1996; Rajabian et al., 2009; Gouin et al., 2010). The intracellular expression of InlC and presence of an LRR domain in this protein suggested that InlC might stimulate *Listeria* protrusions by interacting with a cytoplasmic host target. Such a target, the human scaffolding protein Tuba, was identified through a yeast two-hybrid screen of human cDNAs using InlC as bait (Rajabian et al., 2009).

Tuba is a 177 kDa protein scaffolding protein involved in F-actin assembly (Salazar et al., 2003; Kovacs et al., 2006; Otani et al., 2006), cell junction regulation (Otani et al., 2006), cell morphogenesis (Bryant et al., 2010; Qin et al., 2010; Kovacs et al., 2011), and exocytosis (Bryant et al., 2010; Sato et al., 2012). Tuba comprises several functional domains, including a potentially lipid binding Bar domain, a Dbl homology (DH) domain that activates the GTPase Cdc42, and six Src Homology 3 (SH3) domains (Salazar et al., 2003; Otani et al., 2006) (Figure 1B). The first four SH3 domains in Tuba interact with the GTPase Dynamin, whereas the last SH3 domain (termed “SH3-6”) binds several human ligands, including the actin regulatory protein N-WASP (Salazar et al., 2003). Ligands of the fifth SH3 domain remain to be identified. Importantly, InlC binds to Tuba SH3-6, displacing host N-WASP (Rajabian et al., 2009).

Experiments with polarized Caco-2 BBE1 cells indicate that Tuba limits protrusion formation of Δ*inlC* mutant *Listeria* (Rajabian et al., 2009). Specifically, the defect in protrusions normally observed with this mutant is suppressed when Tuba is depleted through RNA interference (RNAi). Importantly, Tuba depletion has no effect on protrusion formation by wild-type *Listeria* expressing InlC. These genetic data indicate that host Tuba has the potential to impair *Listeria* spread unless bacteria intervene by producing InlC, which antagonizes Tuba. Biochemical and genetic studies indicate that one of the ways that InlC inhibits Tuba is by binding to its SH3-6 domain, thereby disrupting Tuba/N-WASP complexes. Thus, Tuba and N-WASP comprise a host signaling pathway that must be antagonized by InlC in order for *Listeria* to spread efficiently (Figure 1C).

## Role of host Cdc42 in controlling *Listeria* protrusions

The presence of multiple protein or lipid binding domains in Tuba (Figure 1B) prompts the question as to whether Tuba ligands apart from N-WASP affect *Listeria* spread. Interestingly, recent data indicates that the Tuba effector Cdc42 controls bacterial protrusions.

Cdc42 is a human GTPase that regulates many biological processes, including cell motility, endocytic and exocytic trafficking of vesicles, the formation and maintenance of cell junctions, and cell polarity (Jaffe and Hall, 2005; Otani et al., 2006; Harris and Tepass, 2010). The DH domain of Tuba activates Cdc42, without affecting related GTPases (Salazar et al., 2003; Otani et al., 2006). Importantly, *Listeria* actively antagonizes host Cdc42 in order to promote bacterial spread (Rigano et al., 2014). Infection of Caco-2 BBE1 cells causes a ~65% reduction in levels of Cdc42-GTP. In addition, a dominant negative allele of Cdc42 restores normal protrusion formation to Δ*inlC* mutant *Listeria*, whereas constitutively activated Cdc42 inhibits protrusions normally made by wild-type bacteria. These latter findings indicate that the ability to inactivate Cdc42 is required for efficient bacterial protrusion formation. Interestingly, the effect of *Listeria* on Cdc42 is largely independent of InlC, and is instead due to an unidentified bacterial factor. The biochemical mechanism by which this factor antagonizes the host GTPase is presently unknown.

A model for how Tuba, N-WASP, and Cdc42 act together to regulate bacterial spread is presented in Figure 1C. In the absence of InlC, Tuba restrains *Listeria* protrusion formation by using its SH3-6 domain to engage N-WASP and its DH domain to activate Cdc42. Given the ability of Cdc42-GTP to activate N-WASP (Suetsugu and Gautreau, 2012), this GTPase may limit protrusions by acting solely through N-WASP. In such a scenario, simultaneous regulation by SH3-6 and Cdc42 would stimulate N-WASP activity in an additive fashion (Carlier et al., 2000; Suetsugu and Gautreau, 2012). Another possibility is that Cdc42-GTP controls bacterial protrusions through a host effector apart from N-WASP. To date, more than 30 effector proteins are known to interact with the GTP bound form of mammalian Cdc42 (Wallace et al., 2010). Future work may uncover functions for some of these effectors in limiting bacterial spread. Wild-type *Listeria* relieves Tuba-mediated inhibition in protrusions by using InlC to block interaction of the Tuba SH3-6 domain with N-WASP (Figure 1C). In addition, bacteria produce an unidentified factor (“X”) that antagonizes host Cdc42. Through these effects on N-WASP and Cdc42, *Listeria* overcomes the barrier to spread that would otherwise by imposed by the Tuba signaling pathway.

## InlC perturbs host cell junctions

Tuba, N-WASP, and Cdc42 each control the structure of cell-cell junctions in epithelial cells. Specifically, depletion of Tuba or N-WASP by RNAi or dominant negative inhibition of Cdc42 causes normally linear tight junctions to slacken (Otani et al., 2006; Rajabian et al., 2009). It is thought that these curved junctions reflect a role for Tuba, N-WASP, and Cdc42 in producing cortical tension at the plasma membrane. Importantly, infection of Caco-2 BBE1 cells with wild-type *Listeria* alters tight junctions similarly to inhibition of Tuba, N-WASP, or Cdc42 (Rajabian et al., 2009). By contrast, infection with Δ*inlC* mutant bacteria fails to affect junctions. Ectopic expression of InlC in the absence of bacteria is sufficient to induce curved junctions. Finally, expression of a constitutively activated allele of Cdc42 restores normal linear junctions to cells infected with wild-type *Listeria* (Rigano et al., 2014). Collectively, these results indicate that InlC perturbs cell junctions through inhibition of Tuba, N-WASP, and Cdc42. By slackening junctions, InlC likely diminishes cortical tension at the host plasma membrane. Such decreased tension may augment *Listeria* spread by removing an inward force that would otherwise oppose bacterial protrusions. Host Tuba, N-WASP, and Cdc42 can be therefore viewed as a junctional regulatory system that has the potential to restrict bacterial spread. By using InlC to antagonize this regulatory system, *Listeria* circumvents host restriction of spreading (Figure 1D).

## InlC–Tuba interaction affects virulence

Genetic data indicate that interaction of InlC with Tuba is needed for efficient cell-to-cell spread in cultured human cells. An alanine substitution of lysine 173 in InlC (K173A) results in a protein that folds normally, but is partly defective in binding the Tuba SH3-6 domain (Rajabian et al., 2009). Importantly, a *Listeria inlC.K173A* strain expressing the mutant InlC protein is compromised for protrusion formation in polarized Caco-2 BBE1 cells. In addition to interacting with Tuba, InlC binds the host protein IKKα (Gouin et al., 2010). InlC–IKKα interaction inhibits the NFκB signaling pathway, thereby dampening production of host pro-inflammatory cytokines. The K173A mutation in InlC does not effect *Listeria*-mediated inhibition of NFκB, indicating that this mutation specifically uncouples InlC from host Tuba (Leung et al., 2013). Consequently, studies with the *Listeria inlC.K173A* strain indicate that efficient cell–cell spread in cultured cells requires the ability of InlC to bind Tuba. By contrast, regulation of NFκB by InlC likely does not affect bacterial spread.

A recent study took advantage of the *inlC.K173A* strain to address if InlC–Tuba interaction is needed for virulence in a mouse model (Leung et al., 2013). In intravenously inoculated animals, the *inlC.K173A* mutant strain had a 50% lethal dose (LD_50_) that was about 5-fold higher than that of the isogenic wild-type strain. Importantly, this increase in LD_50_ was essentially identical to that observed for a mutant strain deleted for the *inlC* gene (Δ*inlC*). Compared to the wild-type strain, the *inlC.K173A* mutant strain exhibited lower bacterial loads in the liver. Histological analysis of livers indicated that the *inlC.K173A* strain produced smaller foci of infection than did the wild-type strain. These smaller foci are consistent with a role for InlC in cell-cell spread *in vivo*. Taken together, these results provide evidence that interaction of InlC with host Tuba is important for full virulence.

## Structural elucidation of Tuba inhibition by InlC

**Figure 2:**
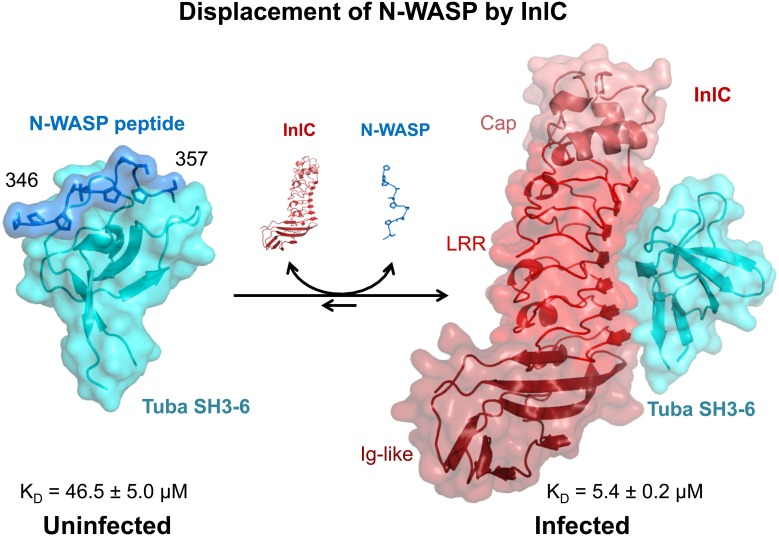
**Molecular Displacement of human N-WASP from human Tuba SH3-6 by InlC of *Listeria monocytogenes*:** the critical physiological interaction of N-WASP with Tuba is mediated by the recognition of a proline-rich peptide of N-WASP (blue, residues 346–357) by a peptide binding groove on the sixth SH3 domain (SH3-6, cyan) of Tuba. This interaction is disrupted by InlC (shades of red: pink—N-terminal Cap domain, red—central leucine rich repeat or LRR domain, maroon—C-terminal Ig-like domain) secreted by *L. monocytogenes* during infection, which binds to a partly overlapping surface of Tuba SH3-6. While the latter interaction is not itself very tight (*K_D_* = 5.4 μM), it is sufficiently tight to outcompete the interaction of N-WASP with Tuba SH3-6 which at *K_D_* = 46.5 μM is roughly an order of magnitude weaker but typical of transient or reversible, physiological interactions.

The molecular and atomic details underlying the displacement of N-WASP from Tuba SH3-6 by InlC (Rajabian et al., 2009) were recently investigated structurally and biophysically (Polle et al., 2014) (Figure 2).

The structural analysis of Tuba SH3-6 with its physiological partner N-WASP, revealed a typical SH3 domain structure for Tuba SH3-6 consisting of a five-stranded, antiparallel β-barrel with a 3_10_-helix in loop β 4–β 5 (Polle et al., 2014). The proline-rich peptide ^346^pppalpssapsg^357^ of N-WASP adopts a polyproline type II (PPII) helical conformation and binds in a class II orientation (Lim et al., 1994) to the peptide binding loop of Tuba SH3-6 located between the conserved loops known as RT-Src (β 1–β 2 loop) and n-Src (β 3–β 4) (Figure 2). The surface area of Tuba SH3-6 and of the peptide involved in the interaction, respectively, amount to ~500 and 600 Å^2^ or 10 and 44% of the respective total. Despite this substantial interaction a dissociation constant of 46.5 ± 5 μM as determined by isothermal titration calorimetry implies a weak and hence transitory interaction. Interestingly, biophysical and crystallographic experiments indicate that the Tuba SH3-6 domain additionally interacts with other Tuba SH3-6 monomers through asymmetric contacts involving two additional parts of its surface distinct from the N-WASP peptide binding groove. Tuba may thus, form higher order structures via this SH3-6 domain (Polle et al., 2014).

A co-crystal structure of Tuba SH3-6 in complex with InlC indicates that InlC actively binds Tuba SH3-6 through the slightly concave surface of its central LRR domain (Polle et al., 2014) (Figure 2). The conformation of InlC is unchanged from that of its apo-structure (Ooi et al., 2006) confirming the remarkable rigidity of the LRR domain as similarly observed for the structurally related proteins InlA (Schubert et al., 2002) and InlB (Niemann et al., 2007). The interaction surface of InlC of ~520 Å is created by the seven-stranded, parallel β-sheet of the LRR and is centered around the aromatic residue phenylalanine 146 in LRR4 but involves interactions from each of the LRR. Comparing three symmetrically independent InlC/Tuba SH3-6 complexes that constitute the asymmetric unit of the crystal unit cell indicate that variability of InlC is largely confined to side-chain conformations.

The three-dimensional structure of Tuba SH3-6 is also largely conserved in its interaction with InlC, implying that binding by InlC does not induce major conformational changes in Tuba SH3-6. The InlC-binding surface of Tuba SH3-6 (570 Å^2^) involves the n-Src loop, the N-terminal residues of the RT-Src loop, the 3_10_-helix and β-strand 5. This binding interface substantially overlaps but is not identical to that of the peptide binding groove. However, residues one to eight of the 12-reside peptide substantially clash with InlC in a superposition such that peptide binding to Tuba SH3-6 is eliminated by InlC binding (Polle et al., 2014).

Surfaces of InlC and Tuba SH3-6 are roughly complementary such that a significant driving force for complex formation appears to be the entropic exclusion of water molecules. Individual interactions involve π –π and CH··π stacking, general van der Waals contacts, hydrogen bonds and two imperfect salt bridges. Overall the interaction does not appear to be optimized for tight binding. Correspondingly the dissociation constant was found to be *K_D_* = 5.4 ± 0.2 μM, indicating a moderately tight interaction. Most importantly the interaction is ~9 times tighter than that between Tuba SH3-6 and N-WASP, easily out-competing the latter. The physiological relevance of the structurally visualized InlC/Tuba SH3-6 interaction was demonstrated by replacing F146 of InlC by alanine, to prevent its interaction with asparagine 1569 of Tuba SH3-6. *In vitro* this substitution alone essentially abrogates binding (affinity reduced 90-fold to *K_D_* = 487 ± 298 μM), while *in vivo* an engineered *Listeria* strain carrying this mutation behaves identically to an *inlC* deletion mutant both in spread of bacteria and in protrusion formation (Polle et al., 2014).

Unexpectedly K173 is not involved in the direct interaction of InlC with the Tuba SH3-6 domain (Polle et al., 2014). Correspondingly *in vitro* analysis of variant InlC^K173A^ by isothermal titration calorimetry did not indicate altered affinity toward Tuba SH3-6 (Polle et al., 2014). The discrepancy between the *in vivo* and *in vitro* importance of this residue remains enigmatic. Resolving this issue may require larger, multi-domain fragments of Tuba being used in the analysis.

## Outstanding questions and future studies

During the structural studies, the Tuba SH3-6 domain was observed to form dimers, tetramers, and octamers *in vitro* while crystals show Tuba SH3-6 forming helical arrays of variable lengths involving identical asymmetric monomer-monomer interactions (Polle et al., 2014). It is not clear whether the two oligomerization events are equivalent or whether oligomerization is physiologically relevant for the full-length protein. We are currently identifying point mutations that will prevent oligomerization of this domain *in vitro* without affecting peptide-ligand binding. *In situ* production of variant proteins could then indicate whether Tuba SH3-6 oligomerization is physiologically relevant.

Another critical unresolved question is what host physiological processes underlie Tuba-mediated regulation of *Listeria* spread? Answering this question is challenging, given the multiple functions of Cdc42 and N-WASP in actin assembly, endocytosis, exocytosis, and cell polarity (Harris and Tepass, 2010; Suetsugu and Gautreau, 2012). Here we suggest two possibilities: First, Tuba, N-WASP, and Cdc42 may restrain *Listeria* protrusions by maintaining actin filaments at cell junctions (Otani et al., 2006; Kovacs et al., 2011). Such filaments act together with myosin II to generate junctional tension (Gomez et al., 2011). Second, Tuba, Cdc42, and N-WASP may indirectly affect cell junctions and *Listeria* spread by promoting the exocytic delivery of host proteins that generate junctional tension. Interestingly, Tuba, Cdc42, N-WASP each localize to the Golgi apparatus, suggesting a potential role for these proteins in exocytosis (Salazar et al., 2003; Matas et al., 2004; Kodani et al., 2009). Future work should elucidate how Tuba, N-WASP, and Cdc42 control *Listeria* spread. Finally, what are the mechanisms that direct the internalization of *Listeria-*containing protrusions? A recent study demonstrated that human casein kinase 1-α (CK1-α) is needed for the resolution of protrusions into vacuoles containing *Listeria* (Chong et al., 2011). Understanding how this kinase promotes protrusion engulfment will likely require the identification of CK1-α substrates involved in bacterial spread. Research in the next decade is anticipated to make considerable progress in understanding how protrusive structures are generated and exchanged between host cells to mediate *Listeria* spread.

### Conflict of interest statement

The authors declare that the research was conducted in the absence of any commercial or financial relationships that could be construed as a potential conflict of interest.
